# Genetic testing of sperm donors in China: a survey of current practices

**DOI:** 10.3389/fendo.2023.1230621

**Published:** 2023-07-14

**Authors:** Chuan Huang, Qi-Lin Wang, Hui-Lan Wu, Zeng-Hui Huang, Xin-Zong Zhang, Wen-Bing Zhu

**Affiliations:** ^1^ Clinical Research Center for Reproduction and Genetics in Hunan Province, Reproductive and Genetic Hospital of CITIC-Xiangya, Changsha, Hunan, China; ^2^ Institute of Reproductive and Stem Cell Engineering, Basic Medicine College, Central South University, Changsha, Hunan, China; ^3^ NHC Key Laboratory of Male Reproduction and Genetics, Guangdong Provincial Reproductive Science Institute (Guangdong Provincial Fertility Hospital), Guangzhou, Guangdong, China

**Keywords:** human sperm bank, sperm donor, genetic testing, genetic counseling, China

## Abstract

**Background:**

The National Health and Family Planning Commission of China (NHFPCC) issued the “Measures for the Management of Human Sperm Banks,” which was revised in 2003 and is still in effect today. One of the standard guidelines is that potential donors undergo laboratory testing to exclude infectious and genetic diseases and karyotype analysis. However, patient demands for donor genetic testing have also increased, and only karyotype analysis to exclude genetic diseases is not sufficient to meet these demands.

**Objective:**

To examine donor genetic screening practices at sperm banks in China and to evaluate the qualifications and skills of genetic counselors at the banks.

**Materials and methods:**

An electronic survey was distributed to twenty-seven sperm banks to examine donor genetic screening practices at sperm banks in China and to evaluate the qualifications and skills of genetic counselors at the banks. Twenty-six human sperm banks responded to a 32-question survey about their current practices related to genetic testing of sperm donors.

**Results:**

The 26 sperm banks reported that all qualified sperm donors undergo karyotype analysis; 22 banks (84.6%) collected three generations of family history from each qualified sperm donor; 10 (38.5%) reported that they attempted to accommodate special requests from donor semen recipients for particular genetic tests. Only 2 of the 26 (7.7%) sperm banks reported that they performed whole-exome sequencing. At all the sperm banks, consent for genetic testing was obtained as part of the overall contract for sperm donors. Nineteen (73.1%) sperm banks had genetic counselors on their staff, while six (23.1%) had no genetic counselors on their staff but had access to genetic counselors at the hospital. Only one (3.8%) sperm bank had no genetic counselors on their staff or at the hospital.

**Conclusions:**

The need for larger scale genetic testing of donors and recipients and an extensive panel of genetic tests specific to the Chinese population. Additionally, professionally trained geneticists must be employed as genetic counsellors so that the results of genetic tests and their implications can be explained to donors.

## Background

**Figure 1:**
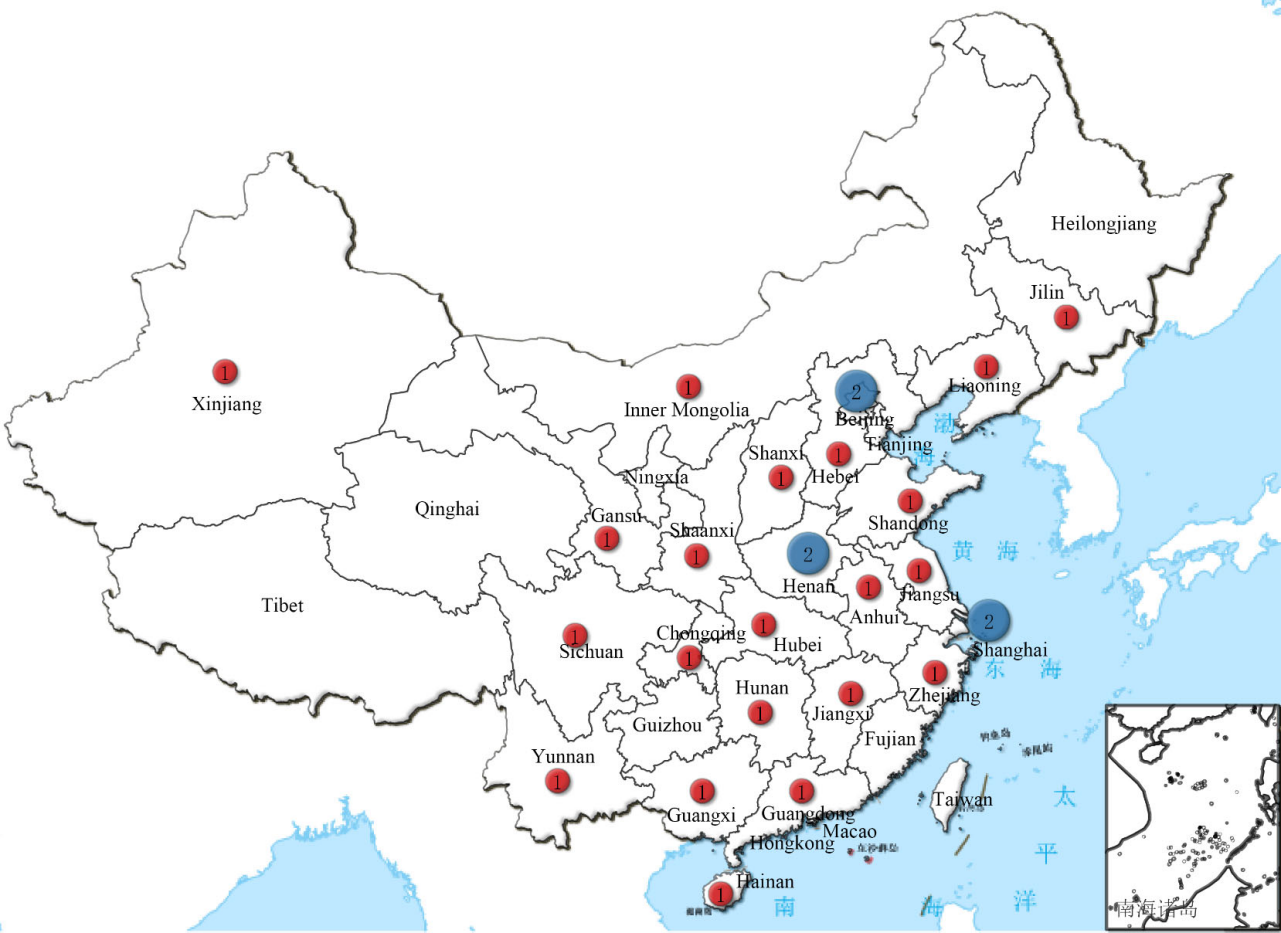
Summary of the geographical distribution of 27 human sperm banks in China.

In early 1981, Lu Hui-Lin and Lu Guang-Xiu established the first human sperm bank in the People’s Republic of China. Despite its unsteady start, human sperm banking has now become a routine part of China’s pervasive and restrictive reproductive complex within a period of 40 years. Today, there are 27 sperm banks in China, and Beijing, Shanghai, and Henan province have two sperm banks each (**Figure 1**).

In 1997, there was a big uproar among professionals and in the media when a donor with 18 donor offspring was found to be suffering from autosomal dominant cerebellar ataxia, which is a severe, untreatable, hereditary neurodegenerative disorder (1). Similarly, in 2002, a Danish sperm donor who was not screened for genetic disorders was found to have neurofibromatosis, and five donor offspring were diagnosed with the condition (2). These incidents point to the need to perform genetic testing in sperm donors. Nowadays, karyotype analysis, three generation family history analysis, specific gene testing (3), genetic carrier screening (4, 5), and whole-exome sequencing (6) are used for testing sperm donors for genetic disorders. In the US, the American Society for Reproductive Medicine (7) and the American College of Medical Genetics (8) recommend that the sperm donor undergo appropriate genetic evaluation, e.g., genetic carrier screening.

In 2001, The National Health and Family Planning Commission of China (NHFPCC) issued the “Measures for the Management of Human Sperm Banks,” which was revised in 2003 and is still in effect today. One of the standard guidelines is that potential donors undergo laboratory testing to exclude infectious and genetic diseases and karyotype analysis (9). Additionally, patient demands for donor genetic testing have also increased, and only karyotype analysis to exclude genetic diseases is not sufficient to meet these demands. Therefore, there appears to be a need to improve the current guidelines to include a larger panel of genetic tests and analyses for donors.

Counseling regarding the residual risk and reproductive implications of genetic testing is best provided by a certified genetic counselor (7). However, to our knowledge, there are no standard guidelines for certified genetic counselors in China. Additionally, all human sperm banks in China are associated with hospitals and do not function as independent facilities. As a result, some human sperm banks in China do not have genetic counselors and may not be able to provide donors with the information they need. This points to the need for more standard guidelines regarding the practices of human sperm banks in China.

In order to provide a comprehensive overview of the genetic testing practices across sperm banks in China, in the present study, we have conducted a survey of human sperm banks in China to examine which genetic tests are performed on sperm donors and have evaluated the professionals involved in family history evaluation and genetic screening of donors.

## Materials and methods

### Study design

This study used a questionnaire to survey the genetic testing of sperm donors in China. The survey consisted of 32 questions that aimed to evaluate the current practices in genetic testing of sperm donors in human sperm banks, including basic information about the sperm banks, the methods used for genetic testing of sperm donors, information about genetic counselors at the sperm bank, and the procedures for managing test results. The completion and submission of the questionnaires by participants indicated their consent for participation in the study.

Study Population and Procedure

Human sperm banks in the People’s Republic of China were identified through an internet search and information on the NHFPCC website. Twenty-seven human sperm banks were identified and invited to participate in this study. Each sperm bank was contacted by telephone or WeChat (a Chinese social media platform) and asked to nominate an individual who would be most suited to answering the online questionnaire on genetic screening practices at the sperm bank. The questionnaire was distributed through WeChat. The individuals who were contacted were asked to forward the survey to an appropriate staff member if they felt that they could not adequately address the survey questions.

All responses were anonymized. One week after the surveys were distributed, the participants were contacted by telephone to remind them of the opportunity to participate if they had not yet had the chance to do so. The study protocol was approved by the Ethics Committee of Central South University approved of this study (approval no. 2021-KT48).

## Results

### Survey respondents

**Figure 2:**
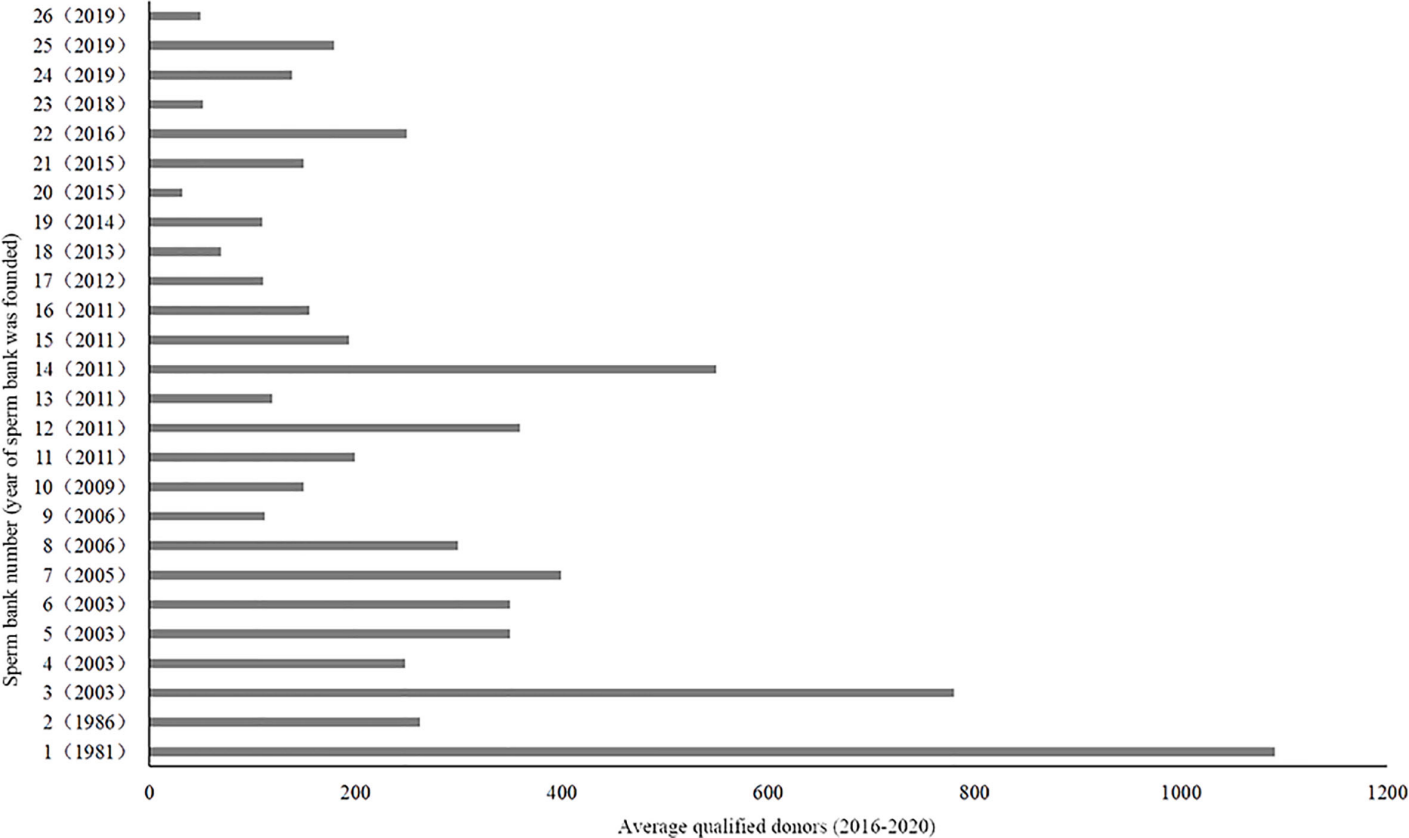
Summary of the 26 participating human sperm banks in China.

Responses were received from 26 of the 27 human sperm banks (96.3%) that were contacted. The individuals who responded were the heads of the sperm banks, as shown in **Figure 2**. The data on the year of establishment of the sperm banks show that the first human sperm bank was established in 1981, and since 2011, there has been a rapid increase in the number of human sperm banks. Currently, 6,000 to 7,000 young men in China become qualified sperm donors each year. During the period of 2016 to 2022, the largest human sperm bank screens out 1092 eligible sperm donors each year, while the smallest only identifies 33.

### Donor genetic testing

The participating sperm banks reported that all qualified sperm donors undergo karyotype analysis. In addition, 22 of the 26 sperm banks (84.6%) collect data on three generations of family history from each qualified sperm donor (**Table 1**). This is in keeping with the NHFPCC guidelines, which recommend that sperm donors undergo karyotype analysis and risk assessment based on their family history. In addition, 10 of the 26 (38.5%) sperm banks reported that they attempted to accommodate special requests from donor semen recipients for particular genetic tests on specific sperm donors, and 5 of the remaining 16 (31.3%) sperm banks reported that they also made provisions to perform specific genetic tests as requested by the recipients within one year. Only 2 of the 26 (7.7%) sperm banks reported that they performed whole-exome sequencing of the sperm donors. Further, 10 of the remaining 24 (41.7%) sperm banks reported that they would also perform whole-exome sequencing on sperm donors within one year.

**Table 1:** Summary of genetic testing performed at human sperm banks in China.

	Karyotype Analysis	Three-generation Family History Analysis	Specific Gene Testing	Plans to Introduce Specific Gene Testing Within One Year	Whole-Exome Sequencing	Plans to Introduce Whole-Exome Sequencing Within One Year
1	√	√	√	/	√	/
2	√	√	√	/		
3	√	√	√	/		√
4	√	√	√	/		√
5	√	√		√		
6	√	√		√		√
7	√	√	√	/		
8	√			√		√
9	√	√				
10	√	√				
11	√					
12	√	√		√		√
13	√	√				
14	√	√	√	/		√
15	√	√				
16	√	√	√	/		
17	√					
18	√	√				
19	√	√	√	/		√
20	√	√				
21	√	√	√	/		√
22	√					
23	√	√				
24	√	√		√		√
25	√	√			√	/
26	√	√	√	/		√
Total	26 (100%)	22 (84.6%)	10 (38.5%)	5 (31.3%)	2 (7.7%)	10 (41.7%)

/: Not applicable.

√: Yes.

The data in **Table S1** illustrate that among the genetic screening tests requested, the majority were for thalassemia-related genes (70%, 7/10), which were closely followed by genes related to deafness (20%, 2/10) and spinal muscular atrophy. In contrast, several of the sperm banks reported that they rarely received requests for such testing, but they still performed specific gene tests on donors based on requests by individual recipients. At the two sperm banks that performed exome sequencing, variations in the GJB2 gene were most frequently identified (**Table S2**): one human sperm bank reported that 8 of 43 donors (18.6%) were carriers of pathogenic variants of GJB2, while the other one reported that 15 of 105 donors (14.3%) were carriers of pathogenic variants of GJB2.

### Genetic counselors

As shown in **Table 2**, 19 of the 26 (73.1%) sperm banks had genetic counselors on their staff, and 6 of the 26 (23.1%) sperm banks had no genetic counselors on their staff but had access to genetic counselors at the hospital. Only one (3.8%) sperm bank had no genetic counselors, even at the hospital. Further, 61.5% of the sperm banks had only one genetic counselor, but 65.4% of the hospitals had two or more genetic counselors. In 73.1% of the hospitals, the genetic counsellor was a doctor, which was the highest level of education reported, while the genetic counsellor was a doctor in only 34.6% of the sperm banks. With regard to the genetics-specific qualifications, some of the counsellors had no formal genetics training while some had advanced degrees in genetics (**Table 2**). Additionally, 61.5% of the genetic counselors were continuing their education in genetics. In all the human sperm banks in China, consent for genetic testing was obtained as part of the overall contract for sperm donors. In 16 of the 26 (61.5%) sperm banks, the sperm bank staff were responsible for obtaining informed consent for the genetic testing of donors; in 6 sperm banks (23.1%), hospital staff were involved in the informed consent process; and in 4 sperm banks (15.4%), both hospital and sperm bank staff were involved.

**Table 2:** Information about genetic counselors at the human sperm banks.

	Number of Human Sperm Banks (n = 26)
Genetic counselors	
Only at the sperm bank	0 (0%)
Only at the hospital	6 (23.1%)
Both at the sperm bank and hospital	19 (73.1%)
No genetic counsellors	1 (3.8%)
Number of genetic counselors at the sperm bank	
0	7 (26.9%)
1	16 (61.5%)
2	3 (11.5%)
>2	0 (0%)
Number of genetic counselors at the hospital	
0	0 (0%)
1	2 (7.7%)
2	3 (11.5%)
>2	17 (65.4%)
No information	4 (15.4%)
Highest level of education of genetic counselors at the sperm bank	
Doctorate	9 (34.6%)
Master’s degree	7 (26.9%)
Bachelor’s degree	3 (11.5%)
No background in genetics	7 (26.9%)
Highest level education of genetic counselors at the hospital	
Doctorate	19 (73.1%)
Master’s degree	2 (7.7%)
Bachelor’s degree	2 (7.7%)
No background in genetics	1 (3.8%)
No information	2 (7.7%)
Extent of genetics training of the genetic counselors	
No formal genetics training	3 (11.5%)
Single genetics course during medical or graduate training	2 (7.7%)
More than one course in genetics	5 (19.2%)
Continuing education in genetics	16 (61.5%)
Staff members who were involved in the informed consent process for genetic testing of donors	
Staff members at the sperm bank	16 (61.5%)
Staff members at the hospital	6 (23.1%)
Staff members at both the sperm bank and the hospital	4 (15.4%)

As shown in **Table 3**, there are inconsistencies when conducting genetic screening practices in China. Specifically speaking, 15 of the 26 (57.7%) sperm banks claimed that they had followed the genetic screening guidelines of at least one professional organization, and 11 sperm banks (42.3%) stand no need to follow any official guidelines. Donors were informed of their genetic test results in person and over the telephone. While 21 sperm banks informed the donor of the results if they were abnormal, 3 of the 26 (11.5%) sperm banks believed that there was no need to inform the donor of the results. Importantly, it was not clear whether donors with mutations of unclear significance mutation were eliminated.

**Table 3:** Items for which there was no consensus between the participating human sperm banks.

Number of Human Sperm Banks (n = 26)	
Guidelines for genetic testing	
Guidelines of the ACMG and ACOG	2 (7.7%)
Standard guidelines of the NHFPCC	12 (46.2%)
Other guidelines	1 (3.8%)
No information	11 (42.3)
Disclosure of the results of genetic testing to donors	
Yes	21 (80.8%)
No (but it is required)	1 (3.8%)
No (and it is not required)	3 (11.5%)
Based on the donor’s choice	1 (3.8%)
Elimination of donors with mutations of unclear significance	
Yes	15 (57.7)
No	3 (11.5%)
Unclear	8 (30.8%)

ACMG, American College of Medical Genetics.

ACOG, American College of Obstetricians and Gynecologists.

NHFPCC, National Health and Family Planning Commission of China.

## Discussion

The data from the present survey show that the prevalence of genetic testing and the protocol for the tests are not standardized and differ across human sperm banks in different regions of China.

In recent years, the cost of molecular genetic tests has greatly reduced due to the emergence of next-generation sequencing technologies, which have allowed for the analysis of a large number of genes and the concurrent evaluation of hundreds of mutations (10). Hence, some reproductive centers and patients have called for the implementation of more extensive genetic screening of sperm donors in human sperm banks. Cryos International is the largest sperm bank in the world that supplies frozen donor sperm to over 100 countries. They employ a bespoke extensive carrier screening panel that screens males for 46 genetic disorders (11). In contrast, none of the human sperm banks in China that participated in this study use carrier screening panels for donors. This is probably because the sperm banks in china recruit donors for Chinese only, more important, there is no bespoke extensive carrier screening panel for Chinese people yet. The carrier frequency of disease-related genes identified at the Translational Medicine Center of Children’s Hospital of Fudan University is more similar for the East Asian population than those for the European population, as reported by the Exome Aggregation Consortium (12). The differences in carrier frequency among different populations indicate the need to establish a panel of genes that is specific to the Chinese population for genetic testing. However, there is also considerable debate within the sperm banking community as to whether sperm donors should undergo expanded carrier screening (4, 13). According to reports from some countries, testing of a higher number of genes meant that fewer donors would be eligible. Additionally, the heads of some sperm banks consider that carrier screening is expensive and considerably adds to the cost of screening a sperm donor. Despite this, carrier screening is a powerful tool, especially in certain high-risk ethnic populations.

Present study showed there are 10 banks in China are using NGS (Next-Generation Sequencing) to test specific genetic now. However, as we know, it cannot be detected by NGS in some specific genes mutations, for example: HBA1, HBA2, SMA etc. Therefore, the gap-PCR method is used for detecting thalassemia-related genes. Also, to our knowledge, the hemoglobin electrophoresis was used for detect thalassemia-related genes in sperm bank 14, 19 and 21, these banks test all donors thalassemia. However, the thing is, different methods would show different detection rates and interpretations. Also, they all have advantages and limitations (14). Therefore, it is very necessary to uniform and standardize the methods of specific genetic testing in human sperm banks.

In the present study, two human sperm banks in China used whole-exome sequencing to screen sperm donor and donor–recipient matches. The GJB2 mutation was the most common pathogenic mutation found in sperm donors. The data from the two sperm banks showed that 18.6% and 14.4% of qualified sperm donors in China were carriers of pathogenic variants of GJB2. This is higher than the carrier frequencies for GJB2 that were previously reported (15, 16). However, this is not surprising, as previous studies on carrier frequencies tested for known mutations, whereas the exome sequencing at these two clinics explored the entire coding region of most genes. Accordingly, the carrier frequencies of GJB2 should not be over-interpreted, as different GJB2 pathogenic variants are associated with various phenotypes of hearing loss. For instance, both p.V37I homozygotes and compound heterozygous p.V37I variants of GJB-2 indicate a significantly higher risk of developing hearing loss. Conversely, heterozygous p.V37I variants alone do not increase the risk of hearing loss (17). Nonetheless, the frequency of GJB2 variants should be a matter of concern in sperm donors in China, along with the frequency of thalassemia- and spinal muscular atrophy-related gene variants. At the time of this study, an increasing number of human sperm banks in China reported that additional genetic tests were under development. Increased public awareness and increasing requests for special tests are likely to influence genetic testing at sperm banks in China in the future.

Genetic counseling is an integral part of genetic testing of sperm donors. In 1996, a study reported that 6 out of 16 (37.5%) sperm banks had a genetic counselor on their staff (18). In 2013, it was documented that 7 of 13 (53.8%) sperm banks already have genetic counsellors, although mostly in part-time positions (19). The present study found that 25 out of 26 (96.2%) sperm banks had genetic counselors on their staff (with six of them in part-time positions), and 20 of the 26 (76.9%) sperm banks had more than one genetic counselor on their staff. This denotes a positive trend in human sperm banks across China. Unfortunately, our data also indicated that not all of the counsellors had adequate qualifications. Comparing with European and American countries, it gets starting late in development of genetic counselor in China. Therefore, there is no standard guidelines for being a qualified genetic counsellors yet. Be specific, it is not difficult of being a genetic counsellor in China. He or she needs to participate some courses and training regardless their previous degrees or background. Also, the organizer could come from folk not official. Even majority of them claimed they have been trained well but normally the course last 2-3 days only.it can be seemed as a gentics-related meetings. In another hand, there is no official paper materials yet also. They could follow any curriculum. In a word, no standardized in China yet. Genetic counselors who are not trained in genetics may not be able to prepare a donor for the results and or be able to provide support to the donor for sharing this information with their at-risk relatives (19). This is probably why some sperm banks in China believed that there was no need to inform the donor of the results. However, the authors believe that it is unethical to withhold the genetic testing results from a donor, as they would be unaware of the risks of bearing progeny in the future. Hence, it is important to have a qualified genetic counselor who is capable of explaining the risks based on the genetic test results and the limitations of each test. A professional genetic counselor would also be able to provide accurate information and obtain the informed consent of sperm donors in the appropriate way. Therefore, it is highly recommended to establish a standardized genetic counseling procedure and training book official as soon as possible, at the same time, the organizer has to be professional enough. Then, more and more qualified counselors could be the staff in banks in the near future.

Genetic testing of sperm donors was introduced in China much after it was initiated in other countries. In the United States, sperm banks frequently cite the guidelines of organizations such as the American Society of Reproductive Medicine (7) and the American College of Medical Genetics (20). In Europe, too, sperm banks have been following the official guidelines of the European Union. In the present study, 12 sperm banks (46.2%) followed the genetic screening guidelines of the NHFPCC, but 11 sperm banks (42.3%) did not follow any standard guidelines. The majority of the sperm banks reported that they are not currently considering additional genetic tests for sperm donors because there are no official guidelines for the genetic evaluation of sperm donors. Hence, it is necessary to develop a consensus on genetic testing of sperm donors in China, so that all sperm banks can benefit from specific and clear practical recommendations with regard to the genetic screening of donors and the disclosure of test results. To achieve this, it is important to engage academics and practitioners in the field of genetics to gather information, discuss, and come to a consensus about the guidelines that must be established. The important questions that need to be answered are: (a) Which genetic tests should be performed on sperm donors? (b) Which genetic disorders should be tested through an extensive carrier screening panel specific for the Chinese population? (c) Should donors with mutations of unclear significance be eliminated? (d) Should the results of the genetic tests be disclosed to donors?

The present study has certain limitations such as survey recipients were asked to forward the survey to an appropriate staff member who would be most suited to answering the online questionnaire, which might have led to self-selection bias. In addition, the sperm bank in some provinces was relatively small, the number of qualified sperm donors was few, hence, and the findings in these sperm banks might have led to bias.

## Conclusions

This is the first study to examine donor genetic screening practices at sperm banks and to evaluate the qualifications and skills of genetic counselors at the sperm banks in China. The results of this study are significant for the development and standardization of sperm banks in China. They imply that genetic tests for donors and recipients, apart from karyotype and three generation family history analysis, must be performed on a wider scale. Additionally, based on data available about birth defects in donor offspring and whole-exome sequencing of donors, an extensive panel of genetic tests specific to the Chinese population must be developed. In addition, a professional geneticist trained in genetic counseling must be employed by sperm banks so that the results of the tests and their implications can be clearly explained to the donors and their corresponding needs can be addressed. These strategies will benefit donors as well as the sperm banks.

## Data availability statement

The original contributions presented in the study are included in the article/supplementary material. Further inquiries can be directed to the corresponding authors.

## Ethics statement

The studies involving human participants were reviewed and approved by Ethics Committee of Central South University. The patients/participants provided their written informed consent to participate in this study.

## Author contributions

Study conceptualization and design, patient recruitment, and data collection: CH, Q-LW, X-ZZ, and W-BZ. Data analysis and manuscript drafting: CH, Z-HH and X-ZZ. Patient recruitment and data collection: H-LW. All authors approved the final version of the manuscript. All authors contributed to the article and approved the submitted version.
