# Supplementary material for: Genetic testing of sperm donors in China: a survey of current practices
Source: Front Endocrinol (Lausanne). 2023 Jul 14;14:1230621. doi: 10.3389/fendo.2023.1230621 (PMC10390298; doi:10.3389/fendo.2023.1230621)
Supplement: Supplementary file 1 [file Table_1.doc]

Table S1. Specific gene testing for disorders in sperm donors

|  | No. of donors | Main genes identified through specific gene testing | Donor population tested |
| --- | --- | --- | --- |
| 1 | 278 | Thalassemia-related genes | Only if requested |
| 2 | 4 | Deafness-related genes | Only if requested |
| 3 | 4 | Spinal muscular atrophy-related genes | Only if requested |
| 4 | 20 | Thalassemia-related genes | Only if requested |
| 7 | 15 | Deafness-related genes | Only if requested |
| 14 | No information | Thalassemia-related genes | All donors |
| 16 | 4 | Thalassemia-related genes | Only if requested |
| 19 | 285 | Thalassemia-related genes | All donors |
| 21 | No information | Thalassemia-related genes | All donors |
| 26 | 2 | Thalassemia-related genes | Only if requested |

Table S2. Whole-exome sequencing for disorders in sperm donors

|  | No. of donors | Major gene variations identified with whole-exome sequencing (%) | Donor population tested |
| --- | --- | --- | --- |
| 1 | 43 | GJB2 (18.6%) | Only if requested |
| 25 | 105 | GJB2 (14.3%) | All donors |
